# Retrospective Cross-Sectional Study Reviewing the Effectiveness of Mycophenolate Mofetil on Saudi Children With Nephrotic Syndrome

**DOI:** 10.7759/cureus.49679

**Published:** 2023-11-29

**Authors:** Deemah A Aldakheel, Abdulrahman Alamir, Khawla K Almarshad, Zahra A Alsafwani, Roa A Alshaer, Abdullah T Alzulfah, Soud A Al Rasheed

**Affiliations:** 1 College of Medicine, King Saud Bin Abdulaziz University for Health Sciences, Riyadh, SAU; 2 Pediatric Nephrology, King Abdullah Specialized Children's Hospital, Riyadh, SAU; 3 Division of Nephrology, Department of Paediatrics, Ministry of National Guard – Health Affairs, King Abdulaziz Medical City, Riyadh, SAU

**Keywords:** steroid-sensitive nephrotic syndrome, immunosuppressive medications, corticosteroid, nephrotic syndrome, mycophenolate mofetil (mmf)

## Abstract

Background

Idiopathic nephrotic syndrome (INS) is the most common form of nephrotic syndrome (NS) in children. It is often associated with minimal change disease (MCD). Corticosteroid therapy is the initial treatment, but many patients experience relapses, leading to steroid-dependent nephrotic syndrome (SDNS) or frequently relapsing steroid-sensitive nephrotic syndrome (FR-SSNS). To avoid prolonged steroid use, mycophenolate mofetil (MMF) is used as an immunosuppressive alternative. MMF is safe and effective for treating SDNS and FR-SSNS in children, with studies showing reduced relapse rates. The current study aims to evaluate MMF's effectiveness and safety in Saudi children with NS and identify factors that affect its efficacy.

Methods

A retrospective cross-sectional study was conducted at King Abdullah Specialized Children's Hospital (KASCH) in Riyadh, Saudi Arabia. The study included children aged one to 14 years diagnosed with NS who received MMF therapy. Data were collected from medical records from 2000 to 2020. Ethical considerations were followed, and statistical analysis was performed using IBM SPSS Statistics for Windows, version 25 (released 2017; IBM Corp., Armonk, New York, United States). Baseline characteristics and responsiveness to MMF were examined.

Results

In our study, 45 participants (25 males, 20 females) with NS were treated with MMF. Most participants were steroid-dependent (84.44%) and had frequent relapses. MMF was effective in 84.4% of cases, with a significant reduction in relapse; the mean number of relapses decreased from 3.5 before MMF to 1.6 after MMF (p-value = 0.00002). Moreover, 40% of the participants were completely free of relapse after the introduction of MMF. The average duration of the MMF therapy was 45 months. Post-MMF side effects were rare but documented. Gastrointestinal symptoms were extremely rare. Elevated liver enzyme levels were reported in 8.88% (four cases) of the participants. Leukopenia, a more common adverse effect, was reported in 26.66% of cases during the MMF therapy. The average daily dose of steroid was reduced from 12.5 mg/day pre MMF to 2 mg/day post MMF with a p-value of 0.00229.

Conclusion

Our study evaluated the use of MMF in 45 participants with NS. We found that MMF was effective in 84.4% of cases, leading to a significant reduction in the number of relapses. Post-MMF side effects were relatively rare, except for leukopenia that was reported in 26.66%. In addition, the average rate of reduction of steroid exposure before and after MMF was significant. These findings suggest that MMF is a promising treatment option for children with NS and an alternative therapy to long-term steroid use, due to its safety and effectiveness, although close monitoring for potential side effects is essential.

## Introduction

The most common form of nephrotic syndrome (NS) in children is idiopathic nephrotic syndrome (INS). It is most often associated with minimal change disease (MCD) upon renal biopsy findings. Although most patients respond to corticosteroid therapy, the relapse course is experienced in about 70%, and some develop steroid-dependent nephrotic syndrome (SDNS) [1-3]. In addition, frequently relapsing steroid-sensitive nephrotic syndrome (FR-SSNS), defined as four relapses or more per year, develops in about 30% of INS patients [4]. Many immunosuppressive medications are used as steroid-sparing agents in SDNS patients to maintain remission instead of using corticosteroids for longer periods of time and exposing them to steroid toxicity. Mycophenolate mofetil (MMF), a non-nephrotoxic immunosuppressive agent, the prodrug of mycophenolic acid, blocks de novo purine synthesis and inhibits the proliferation of T and B lymphocytes, antibody production, and cytokine gene expression [5]. The major advantages of MMF are the absence of steroid toxicity and nephrotoxicity [6]. A study was conducted in Saudi Arabia, by Al-Akash on 2005, to evaluate the safety and efficacy of MMF in treating pediatric patients with SDNS or FR-SSNS [7]. They retrospectively reviewed the medical records of 18 patients, and 11 were included. The results showed that MMF is a safe and effective option for the treatment of children with SDNS or FR-SSNS [7]. A 2007 study that assessed the management of MMF and prednisolone for SDNS in Saudi Arabia found that the relapse rate after introducing MMF significantly decreased [8]. The aim of our study is to evaluate the effectiveness of MMF in Saudi children with NS and its safety measures and identify the factors that influence its efficacy.

## Materials and methods

This retrospective cross-sectional study was conducted at King Abdullah Specialized Children’s Hospital (KASCH), Ministry of National Guard Health Affairs, Riyadh, Saudi Arabia. Data were collected from the medical records of children aged one to 14 years, followed up for NS in the pediatric nephrology outpatient clinic at KASCH between 2000 and 2020. The following data were collected: clinical data (gender, current age, and age at diagnosis of NS), personal and medical history, and previous use of other therapies. The study sample consisted of both male and female patients.

The inclusion criteria were as follows: patients aged one to 14 years, patients diagnosed with NS, and patients undergoing MMF therapy. The exclusion criterion was patients aged over 14 years or under one year.

The sample size was calculated using EpiTools Epidemiological Calculators (EpiTools, www.epitools.ausvet.com.au) [9]. Non-probability purposive sampling was used to identify patients who fit the criteria. Informed consent was waived because chart review studies involve no patient contact. Patient confidentiality was maintained throughout the study; no names or medical record numbers were mentioned (serial numbers were used instead). The data were always stored on password-protected computers and was only accessible to team members. Data collection was carried out after receiving grant approval from the King Abdullah International Research Center.

IBM SPSS Statistics for Windows, version 25 (released 2017; IBM Corp., Armonk, New York, United States) was used to analyze the data. Initially, a univariate analysis using the chi-squared test was performed to examine responsiveness to MMF according to the patients’ background characteristics. In addition, a chi-squared test was conducted to examine the effectiveness of MMF and the factors that influence it. The associations between MMF use and some unfortunate complications of NS, such as thrombosis, renal failure, and infection, were also examined. The safety profile of MMF is described in the form of percentages. The average daily dose of steroid assessed using the Mann-Whitney test. A confidence interval (CI) of 95% and an alpha of 0.05 were used.

Baseline clinical data, serum examination (platelets, white blood cells, neutrophils, lymphocytes, alanine transaminase, aspartate aminotransferase, and creatinine), and urine analysis (protein in urine and red blood cells in urine) were obtained within three months before and after the MMF therapy starting date. Histopathological findings, from the date of diagnosis to 2020, were acquired. Short stature was defined as a height of <third percentile for age and gender. Body mass index was calculated using the formula weight/(height)2 kg/m2. Obesity was defined as a body mass index >95th percentile for age and gender. Hypertension in children was defined as a blood pressure (BP) of >95th percentile for sex, age, and height or taking medication for high BP. The definition of SDNS used was having a relapse of NS within 14 days (about two weeks) of stopping prednisone or after tapering the dosage. Responsiveness to MMF was defined as remission after the use of MMF or a decrease in the number of relapses, whereas complete remission was defined as no more relapses after the initiation of MMF. Relapses were defined by the presence of >+1 protein in urine in three consequent dipstick or urine analyses or the appearance of edema [10].

## Results

Table 1 presents the background characteristics of the participants and the histopathological features of the lesions. It also compares these variables according to their responsiveness to MMF. Our study included 25 males (55.6%) and 20 females (44.4%), with a mean age of 11.6 years. The study participants were diagnosed at a mean age of 3.8 years, started MMF at a mean age of 6.5 years, and had the disease for a mean of 7.3 years. None of these background characteristics showed notable differences with respect to responsiveness to MMF (p > 0.05). Regarding the histopathological findings, 10 cases (22.22%) showed minimal change features, and two cases (4.44%) showed focal segmental glomerulosclerosis. However, for most of the patients, histopathologic testing was not performed, rendering these comparisons unreliable. Moreover, the average dose of MMF was 673 mg/m2 among responsive cases and 679.5 in non-responsive cases; however, these numbers were not statistically significant.

**Table 1:** Comparison of the effectiveness of MMF among genders, ages, age of diagnosis, and duration of disease and histopathology findings. MMF: mycophenolate mofetil, MCD: minimal change disease, SD: standard deviation

Term	Overall n (%)	MMF responsive n (%) (n = 37)	MMF non-responsive n (%) (n = 8)	p value
Gender				0.408
Female	20 (44.44)	18 (48.6)	2 (25)	
Male	25 (55.56)	19 (51.4)	6 (75)	
Renal histopathology				0.219
C1q nephropathy	1 (2.22)	1 (2.7)	0 (0)	
Focal segmental glomerulosclerosis	2 (4.44)	2 (5.4)	0 (0)	
MCD	10 (22.22)	9 (24.3)	1 (12.5)	
Membranous nephropathy	1 (2.22)	0 (0)	1 (12.5)	
Non-applicable (NA)	31 (68.89)	25 (67.6)	6 (75)	
p-values obtained from Pearson’s chi-squared test of independence				
Term	Overall mean (SD)	MMF responsive	MMF non-responsive	p-value
Age (years)	11.6 (4.2)	11.7 (4.2)	10.9 (4.5)	t: 0.6303
Age at Dx (years)	3.8 (2.2)	3.9 (2.2)	3.5 (2.4)	t: 0.6961
Duration of disease (years)	7.3 (4.1)	7.4 (4.2)	6.7 (3.9)	t: 0.7036
Age at starting MMF (years)	6.5 (3.2)	6.6 (3.2)	6.2 (3.6)	t: 0.7761
Dose of MMF mg/m^2^	674.9 (335.7)	673.8 (302.3)	679.5 (488.3)	t: 0.9757
α = 0.05				
p-values obtained from two-sample t-test (t) or Mann–Whitney test (U)				

Table 2 shows the variables featuring MMF effectiveness. Most of the study participants were steroid-dependent (84.4%) and had frequent relapses (73.3%). Overall, the duration of steroid therapy averaged 5.5 years among the study sample. Interestingly, the mean number of relapses was 3.5 before MMF versus 1.5 after MMF. Among the MMF-responsive cases, the mean number of relapses dropped from 3.7 to only 0.9 after MMF.

**Table 2:** Effectiveness of mycophenolate mofetil (MMF) and factors that influence its efficiency MMF: mycophenolate mofetil, N/A: not applicable, SD: standard deviation

Term	Overall n (%)	MMF responsive n (%) (n = 37)	MMF non-responsive n (%) (n = 8)	p-value
Steroid dependent				0.783
No	7 (15.56)	5 (13.5)	2 (25)	
Yes	38 (84.44)	32 (86.5)	6 (75)	
Frequent relapses				0.447
No	10 (22.22)	7 (18.9)	3 (37.5)	
Yes	33 (73.33)	28 (75.7)	5 (62.5)	
N/A	2 (4.44)	2 (5.4)	0 (0)	
MMF type				0.875
MMF oral suspension	12 (26.67)	10 (27)	2 (25)	
MMF tablet	19 (42.22)	15 (40.5)	4 (50)	
NA	14 (31.11)	12 (32.4)	2 (25)	
α = .05				
p-values obtained from Pearson’s chi-squared test of independence				
Term	Overall mean (SD)	MMF responsive	MMF non-responsive	p-value
Duration of steroid Tx (yrs)	5.5 (4.2)	5.7 (4.2)	4.5 (4.5)	t: 0.5117
Number of relapses before MMF	3.5 (1.9)	3.7 (1.8)	2.5 (2.4)	t: 0.2127
Number of relapses after MMF	1.6 (2.3)	0.9 (1)	4.4 (3.9)	t: 0.0394*
Duration of MMF (months)	45 (37.4)	46.2 (39.3)	39.1 (26.2)	t: 0.5637
α = 0.05. p < 0.05*				
p-values obtained from the two-sample t-test (t)				

Regarding the effectiveness of MMF, it was found that 84.4% of the population was responsive. Interestingly, the average number of relapses dropped from 3.5 to 1.6 (p = 0.00002). The results are presented in Tables 3 and 4.

**Table 3:** Average number of relapses before and after MMF therapy. MMF: mycophenolate mofetil

Average number of relapses before and after MMF therapy			
Term	After MMF	Before MMF	p-value
Average number of relapses	1.6	3.57	0.00002 ***
α = 0.05, p < 0.001***			
p-values obtained from t-test (t)			

**Table 4:** Number of responsive and non-responsive cases

Term	Overall N (%)
Non-responsive	7 (15.56)
Responsive	38 (84.44)

Table 5 shows some unfortunate complications of NS, such as thrombosis, renal failure, and infection. The rates of thrombosis and renal failure were low (both <9%), while infections were the most common complication (17.78%).

**Table 5:** Nephrotic syndrome complications

Term	Overall N (%)
Thrombosis	
No	43 (95.56)
Yes	2 (4.44)
Renal failure	
No	43 (95.56)
Yes	2 (4.44)
Infection	
No	37 (82.22)
Yes	8 (17.78)

Table 6 summarizes the safety profile of MMF. Among the documented post-MMF side effects, some were extremely rare, such as include nausea, vomiting, abdominal pain, and diarrhea. Abnormally elevated levels of liver enzymes were reported in four cases (8.88%). A more commonly reported adverse effect was leukopenia, which was reported in 26.66% of cases during MMF therapy.

**Table 6:** Safety of mycophenolate mofetil (MMF)

Variable	N (%)
Gastrointestinal symptoms (nausea, vomiting, abdominal pain, diarrhea)	
No	43 (95.6)
Yes	2 (4.4)
High liver enzymes	
No	41 (91.11)
Yes	4 (8.89)
Leukopenia within three months of MMF introduction	
No	38 (84.44)
Yes	7 (15.56)
Leukopenia during MMF therapy	
No	33 (73.33)
Yes	12 (26.67)

Tables 7-9 show the product of daily dose of steroid. Notably, the average daily dose significantly decreased from 12.5 mg to 2 mg (p = 0.0029). Steroid daily dose adjustment after MMF initiation was assessed and showed that about 38% withdrew from steroidal treatment. In addition, 33% of the cases had their daily steroid dose adjusted to lower doses. Some of the monitored side effects were not of concern, as they only occurred in one to three cases, including gastrointestinal (GI) symptoms, musculoskeletal symptoms, high plasma glucose levels, and cataracts. However, 20% of the study participants developed hypertension post-steroid therapy. In addition, 15.56% of the study sample developed features of short stature post-steroid therapy.

**Table 7:** Mean dose of steroid before and after mycophenolate mofetil (MMF) therapy.

Term	Steroid dose before MMF (mg/day)	Steroid dose after MMF (mg/day)	p-value
Median	12.5	2	0.0229*
α = 0.05, p < 0.05*			
p-value obtained from a Mann–Whitney test			

**Table 8:** Steroid dose adjustment after MMF initiation MMF: mycophenolate mofetil, N/A: not applicable

Steroid daily dose adjustment after MMF initiation	
Term	N (%)
Decreased steroid dose	15 (33.33)
Stopped steroid	17 (37.78)
Increased steroid dose	4 (8.89)
N/A	9 (20)

**Table 9:** Side effects of steroids

Variable	
GI symptoms	
Abdominal pain	1 (2.22)
Gastroenteritis	2 (4.44)
None	42 (93.33)
Short stature	
No	38 (84.44)
Yes	7 (15.56)
Musculoskeletal symptoms	
Bone pain	1 (2.22)
Mineral bone disease (osteoporosis)	1 (2.22)
None	43 (95.55)
HPT	
No	36 (80.00)
Yes	9 (20.00)
High glucose	
No	42 (93.33)
Yes	3 (6.67)
Eye (cataract)	
No	44 (97.78)
Yes	1 (2.22)
Obesity	
No	39 (86.67)
Yes	6 (13.33)

## Discussion

Our data show that gender, current age, age at diagnosis, duration of the disease, and duration of start of MMF therapy had no significant effect on responsiveness to the treatment. MMF was found to be effective in reducing the number of relapses, as the mean number of relapses before MMF therapy was 3.5, which decreased to a mean of 1.6 relapses after MMF in MMF-responsive patients (p < 0.001). Interestingly, 40% of the patients were free of relapses after the initiation of MMF therapy. 

The possibility of prolonging the period of remission in children with SDNS since the early experiences of MMF usage has been confirmed in several observations [3,11,12]. Studies worldwide have examined the efficacy of MMF and looked for predictors of response to therapy in retrospective studies and randomized clinical trials. In a retrospective study with a sample of 96 patients, 48 were able to withdraw from steroids over the duration of 18 months; however, out of those 48, only 26 (30.9%) did not relapse while receiving MMF alone, and of those patients, only six were still in remission after discontinuation of treatment, which indicates that MMF has minimal remnant effects [13]. In our study, 40% of the patients were relapse free after MMF and 84.56% were responsive to MMF; the relapse rate was notably reduced, with the mean duration on MMF being 45 months and the duration of steroid therapy being about 5.5 years on average.

A retrospective study with a 10-year follow up and a sample of 44 patients who were treated with MMF at a median age of 13.3 years conducted in Japan by Fujinaga et al. found that only four patients remained in relapse-free remission when switched from cyclosporine to MMF [14]. At the follow-up, 15 of the patients (34.09%) were in remission and needed no treatment, which is similar to our percentage of 40%. A study conducted in India by Karunamoorthy et al. that assessed the efficacy and safety of MMF found that 83% of the patients were MMF sensitive and were able to achieve remission for five months but relapsed afterward. In our study, the percentage of patients who had two or more relapses before MMF was about 86.67%, which decreased to 33.28% after the initiation of MMF.

Benefits/limitations of MMF versus steroids 

Patients with NS often require steroids and other immunosuppressive medications to maintain remission. In our sample, 38 out of 45 patients (84.44%) were steroid-dependent, and 33 out of 45 (73.33%) frequently relapsed. Although corticosteroids are the mainstay of treatment in children with NS, their prolonged use results in several adverse events that necessitate other therapeutic options to prevent toxicity. Several researchers have reported the effects of prolonged use of steroids, including bone changes, insulin resistance, obesity, hypertension, and vision abnormalities [15-18].

The most reported side effects of steroids in our study were hypertension (20%), short stature (15.56%), obesity (13.33%), high glucose (6.67%), and gastroenteritis (4.44%). Less reported side effects that were only seen in one patient out of the sample include bone pain, cataracts, mineral bone disease, and abdominal pain. Interestingly, the average daily dose of steroid was significantly reduced from an average of 12.5 mg/day before MMF to an average of 2 mg/day after MMF (p = 0.00229), with an average duration of 5.5 years. A 2009 study conducted by Kyrieleis et al. examined the long-term effects of steroids in 78 individuals who were 16 years or older, diagnosed with NS in childhood, and receiving steroid therapy with prednisone 60 mg/m2/day for six to eight weeks, 40 mg/m2/day during the subsequent four to six weeks, and 60 mg/m2 during relapses [17]. The study evaluated the presence of hypertension as a complication of steroids in adults who were treated with steroids during childhood due to NS, and six patients out of 15 were still being treated with antihypertensive medication. 

The most prevalent steroid complication in our sample was hypertension; nine subjects were treated with antihypertensive medication, two of which stopped their antihypertensive agents after the initiation of MMF. In addition, three of our subjects developed high HgbA1c levels while on steroid therapy, one of which had to stop prednisolone due to persistent insulin resistance. In summary, prolonged steroid therapy is associated with many adverse events, which makes finding an alternative medication, especially in the case of diseases requiring longer periods of treatment, very appealing. In our study, about 37% of the participants were able to withdraw from steroid therapy after MMF introduction. In addition, 30% of the cases had their steroid regimens adjusted to lower doses, which makes MMF an attractive alternative to long-term exposure to steroidal therapy in NS patients.

Factors that affect MMF efficacy

Fujinaga et al. found that younger of onset and steroid dependency during treatment are both positively associated with active nephrotic disease in adulthood, whereas Dehoux et al. found that patients with shorter flare episodes, with a shorter disease duration, and who were younger when MMF therapy was initiated were significantly more likely to respond to the treatment, which entailed a reduction in steroid dose and a decrease in relapses [13-14]. A study conducted by Karunamoorthy et al. in India concluded that the risk of MMF failure was significantly higher in patients with late-onset NS [19]. In our study, gender, age, age at diagnosis, and duration of therapy showed no correlation with responsiveness to MMF therapy. The histopathology of NS could be a factor that affects response to treatment; however, most of our sample (68.89%) did not undergo histopathological diagnosis. Only 14 subjects had gone through histopathologic testing, and the biopsy findings revealed that two patients had focal segmental glomerulosclerosis; both were non-responsive to MMF (one was noncompliant, possibly due to several side effects developed during therapy and ended up requiring dialysis to control disease progression, and the other experienced several therapy complications, went into renal failure, and died at the age of 17 years). In addition, 10 patients had MCD, nine of whom were responsive; however, these findings were not statistically significant. Lastly, one of our study subjects had a biopsy that indicated membranous nephropathy; however, the disease did not improve with MMF therapy. Due to the small number of patients who underwent histopathological testing, conclusions could not be generalized. As for NS complications in our sample, 4.4% of the patients developed thrombosis, 4.4% developed renal failure requiring dialysis, and 17.78% developed recurrent infections. These complications did not show any significant effect on responsiveness to MMF therapy.

Dosage and route of MMF 

In our study, the average dose of MMF used was 674 mg/m2 daily and that was evidently effective in reducing the number of relapses in our population. We found that there was no effect of the MMF formulation (suspension or tablet form) on the responsiveness or number of relapses. A study by Ravani et al. used a slightly lower-dose MMF to induce remission in children with NS (350 mg/m2 twice daily); however, this was inadequate for either inducing remission or reducing relapses [20]. Gellermann assessed the difference in the response rate between pediatric patients with NS and divided the participants into two groups, with one receiving a lower dose of MMF than the other. The group with lower MMF exposure experienced a significantly higher number of relapses with shorter periods between them; moreover, the adverse events were not related to the level of MMF exposure [21]. Lastly, a randomized clinical trial conducted in Italy compared the efficacy of rituximab with low-dose MMF in a sample of 30 patients; regrettably, the study had to be stopped due to significantly high rates of relapses in patients receiving low-dose MMF [20]. In our study, the dose that was safely used and successfully reduced the number of relapses in our sample was, on average, 674 mg/m2, as our results show that 84.44% of the study participants had a lower relapse rates while on MMF than before. In addition, in Mendizabal et al.’s study, the mean dose that caused remission in nine out of 21 cases was 624 mg/m2 per 12 hours, and it had fewer undesirable side effects when compared steroidal or other immunosuppressive medication [22]. 

MMF safety profile 

The most notable side effects of MMF in our patient sample with NS were leukopenia, recurrent infections, and elevated liver enzymes. Leukopenia occurred over the course of taking MMF in 26.67% of the sample. More specifically, 15.56% of the sample developed leukopenia after being on MMF within the first three months, three of the patients who experienced leukopenia had their doses of MMF reduced, and MMF was stopped in one patient who was followed up and whose white blood cells returned to normal. Eight patients (17.78%) had recurrent infections that could be attributed to MMF use or NS progression. Although the other most reported adverse effect in our research was elevated liver enzymes (in 8.89% of the sample), these measures were corrected spontaneously when repeated later. In Karunamoorthy et al.’s study, 88% of the sample had no adverse effects as a result of the therapy compared to 62.2% in our study, which makes MMF an attractive choice due to its safe profile [19]. In Karunamoorthy’s study, the 22% of patients who had adverse effects mainly experienced urinary infections, diarrhea, and leukopenia, all of which resolved after stopping MMF therapy. Their study also found that some patients developed features of MMF resistance. In Gellermann et al.’s study, the most frequently reported side effects of MMF were infections; 20 patients reported experiencing acute respiratory tract infection, bronchitis, or otitis media; seven cases had episodes of enteritis; and hyperemesis and loss of appetite were reported in four patients [20]. In our sample, neither nausea nor vomiting was reported in any of the participants; however, abdominal pain and diarrhea were reported in 2.22% of the sample. In 2006, Hogg et al. studied MMF in 32 children with frequently relapsing NS, and leukopenia was observed in five children [22]. 

This study has several limitations. First, the lack of kidney biopsy in some subjects hindered comparison with histopathologic subtypes of NS. Second, the small sample size limited the generalizability of the findings. Lastly, missing data due to paper-based documentation and patients receiving care at different centers compromised the completeness and accuracy of the analysis.

## Conclusions

Our findings highlight the efficacy of MMF in managing NS, as most patients experienced positive treatment outcomes. However, it is important to closely monitor white blood cell counts due to the observed incidence of leukopenia. MMF is an attractive alternative for steroid-dependent NS children, as our results show that subsequent reductions in daily steroid exposure prevent complications associated with their long-term use.
